# PI3K-Akt-mTOR axis sustains rotavirus infection via the 4E-BP1 mediated autophagy pathway and represents an antiviral target

**DOI:** 10.1080/21505594.2017.1326443

**Published:** 2017-06-01

**Authors:** Yuebang Yin, Wen Dang, Xinying Zhou, Lei Xu, Wenshi Wang, Wanlu Cao, Sunrui Chen, Junhong Su, Xuepeng Cai, Shaobo Xiao, Maikel P. Peppelenbosch, Qiuwei Pan

**Affiliations:** a Department of Gastroenterology and Hepatology, Erasmus MC-University Medical Center, Rotterdam, The Netherlands; b Medical Faculty, Kunming University of Science and Technology, Kunming, P. R. China; c State Key Laboratory of Veterinary Etiological Biology, Lanzhou Veterinary Research Institute, Chinese Academy of Agricultural Sciences (CAAS), Lanzhou, P. R. China; d State Key Laboratory of Agricultural Microbiology, College of Veterinary Medicine, Huazhong Agricultural University, Wuhan, P. R. China

**Keywords:** autophagy, intestinal organoids, PI3K-Akt-mTOR-4E-BP1 pathway, rotavirus

## Abstract

Rotavirus infection is a major cause of severe dehydrating diarrhea in infants younger than 5 y old and in particular cases of immunocompromised patients irrespective to the age of the patients. Although vaccines have been developed, antiviral therapy is an important complement that cannot be substituted. Because of the lack of specific approved treatment, it is urgent to facilitate the cascade of further understanding of the infection biology, identification of druggable targets and the final development of effective antiviral therapies. PI3K-Akt-mTOR signaling pathway plays a vital role in regulating the infection course of many viruses. In this study, we have dissected the effects of PI3K-Akt-mTOR signaling pathway on rotavirus infection using both conventional cell culture models and a 3D model of human primary intestinal organoids. We found that PI3K-Akt-mTOR signaling is essential in sustaining rotavirus infection. Thus, blocking the key elements of this pathway, including PI3K, mTOR and 4E-BP1, has resulted in potent anti-rotavirus activity. Importantly, a clinically used mTOR inhibitor, rapamycin, potently inhibited both experimental and patient-derived rotavirus strains. This effect involves 4E-BP1 mediated induction of autophagy, which in turn exerts anti-rotavirus effects. These results revealed new insights on rotavirus-host interactions and provided new avenues for antiviral drug development.

## Introduction

Rotavirus is a member of the *Reoviridae* family.^1^ As the leading cause of severe dehydrating diarrhea, it mainly attacks children younger than 5 y old, resulting in approximate 114 million diarrheal episodes and 453,000 infant deaths annually.^2,3^ Besides children, accumulating evidences indicate that rotavirus could also cause chronic infection in organ transplantation patients, irrespective to the age of the patients.^4-6^ Although 2 safe licensed vaccines named RotaTeq (Merck and Co., PA, USA) and Rotarix (GSK Biologicals, Rixensart, Belgium) are available,^1^ the implementation of these vaccines in many developing countries remains challenge due to high cost and logistic issues.^7^ Since no approved medication is available, the development of effective antiviral therapies is indispensable to combat this pathogen.

Numerous signaling pathways play important roles in regulating virus infection either by supporting or defending the infection of virus.^8^ Phosphatidylinositol 3-kinase (PI3K)-protein kinase B (Akt)-mammalian target of rapamycin (mTOR) axis takes a vital part in regulating various cellular functions and biologic processes, including protein synthesis, cell cycle progression, cell survival, apoptosis, angiogenesis and drug resistance.^9^ In this signaling, PI3K serves as a key node, which is capable of monitoring panels of biologic processes via phosphoryl transfer.^10^ PI3K could be activated by many types of cellular stimuli, which subsequently phosphorylates the inositol PIP2 to PIP3 that recruits Akt to the plasma membrane by binding to its N-terminal pleckstrin homology (PH) domain in which Akt gets activation via phosphorylation.^11^ As a serine/threonine kinase, phosphorylated Akt is able to stimulate mTOR which belongs to a member of the serine/threonine phosphatidyl inositol 3′ kinase-related kinase family (PIKK).^10^

mTOR is an evolutionarily conserved Serine/Threonine kinase playing indispensable roles in regulating mRNA translation, autophagy machinery and cell proliferation.^12^ Furthermore, mTOR can directly activate a variety of cellular effectors such as p70S6 kinase (p70S6K) and eukaryotic translation initiation factor 4E-binding protein 1 (4E-BP1) to control cell growth through integrating nutritional information and receptor tyrosine kinase signaling.^13^ For instance, mTOR controls capped-dependent translation of both cells and viruses via inducing phosphorylation of 4E-BP1 to promote the formation of a functional eukaryotic initiation factor 4F (eIF-4F) complex.^12,14^ The eIF-4F complex comprises 3 subunits including a RNA helicase eIF4A, a cap-binding protein eIF4E and a scaffolding protein eIF4G.^15^ Most of the 4E-BPs share a canonical eIF4E-binding motif (4E-BM) of sequence YX4LΦ with eIF4G (where Y denotes Tyr, X denotes any amino acid, L denotes Leu, and Φ denotes a hydrophobic residue). But some 4E-BPs contain non-canonical 4E-BMs connected by a linker (15–30 residues), and the motifs are not conserved and not required for eIF4G binding.^15^ Autophagy machinery, as another important downstream element of the mTOR signaling, is a cellular non-specific, bulk degradation process involved in removing damaged macromolecules and organelles to defend nutrient and environmental stress,^16,17^ which is also involved in regulating virus infection.^18^

PI3K-Akt-mTOR pathway as a potential antiviral target is involved in the infection of a broad spectrum of viruses including lymphocytic choriomeningitis virus (LCMV),^19^ Middle East Respiratory Syndrome Coronavirus (MERS-CoV),^20^ HIV ^21^ and human cytomegalovirus.^22^ In this study, we comprehensively studied the role of PI3K-Akt-mTOR signaling in rotavirus infection and explored the avenues of therapeutic targeting. Besides conventional 2 dimension (2D) culture based immortalized cells, we also used 3 dimension (3D) cultures of primary human intestinal organoids for modeling rotavirus infection.^23^ These organoids can recapitulate most if not all aspects of *in vivo* tissue architecture of intestinal epithelium.^24^ We found that PI3K-Akt-mTOR pathway is crucial in sustaining rotavirus infection via its downstream effector 4E-BP1 and the induction of autophagy. Importantly, targeting this pathway by pharmacological inhibitors/FDA approved drugs potently inhibited rotavirus infection, providing novel therapeutic approaches for combating this virus.

## Results

### Inhibition of PI3K signaling potently inhibits rotavirus

**Figure 1.**
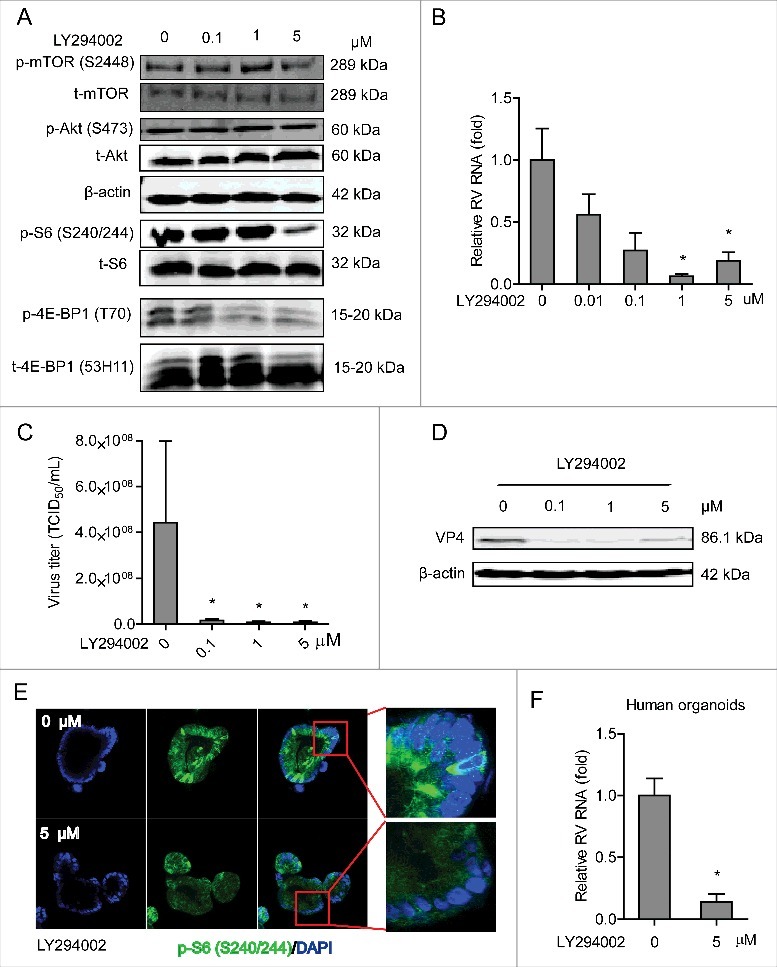
Inhibition of PI3K signaling potently inhibits rotavirus infection. (A) Western blot analysis of p-mTOR (S2448), t-mTOR, p-Akt (S473), p-S6 (S240/244) and p-4E-BP1 (T70) protein levels and the corresponding total protein levels under the treatment of indicated concentrations of LY294002 (48 h) in Caco2 cells. (B) Treatment with LY294002 (48 h) potently inhibited viral genomic RNA in SA11 rotavirus infected Caco2 cells measured by qRT-PCR (n = 4, mean ± SEM, *P < 0.05, Mann-Whitney test). (C) Effects of LY294002 on the production of infectious viral particles determined by TCID_50_ method. Each bar represents the TCID_50_/mL (mean ± SEM) (n = 5, *P < 0.05, **P < 0.01, Mann-Whitney test). (D) Treatment with LY294002 (48 h) potently inhibited viral VP4 protein in SA11 rotavirus infected Caco2 cells determined by western blot assay. (E) Representative confocal immunostainings of p-S6 (S240/244) (Green) after 48 h incubation with 0 (as control) and 5 μM LY294002 in human intestinal organoids. Nuclei were visualized by DAPI (blue). (F) Treatment with LY294002 (48 h) significantly inhibited viral genomic RNA in SA11 rotavirus infected human intestinal organoids examined by qRT-PCR (n = 4, mean ± SEM, *P < 0.05, Mann-Whitney test).

Since PI3K serves as a key node in the PI3K-Akt-mTOR pathway, its effect on rotavirus was tested. LY294002 is a potent inhibitor of PI3K. In human Caco2 cells, treatment with 5 μM LY294002 potently inhibited phosphor-Ser-240/244 S6 and phosphor-4E-BP1 (T70) but not the corresponding total proteins (as controls) (Fig. 1A and Figure S1A). Importantly, treatment with 1 and 5 μM LY294002 for 48 h resulted in 93.5 ± 1.7% (n = 4; *P* < 0.05) and 81.0 ± 6.8% (n = 4; *P* < 0.05) reduction of viral RNA, respectively (Fig. 1B). LY294002 could also significantly suppress rotavirus RNA secretion (Figure S2B). The inhibitory effect of this compound was further verified by TCID_50_ method, demonstrating that it could remarkably inhibit the production of infectious viral particles (Fig. 1C). Western blot assay further confirmed that treatment with 0.1, 1 and 5 μM LY294002 could vigorously inhibit rotavirus protein synthesis in Caco2 cells (Fig. 1D). The effect of this drug was also validated in human primary intestinal organoids. Immunofluorescent staining showed that phosphor-Ser-240/244 S6 was potently inhibited by treatment of 5 μM LY294002 (Fig. 1E), supporting that human intestinal organoid is also a PI3K signaling proficient model. Consistently, LY294002 inhibited rotavirus infection in organoid model. For instance, 5 μM LY294002 resulted in 86.0 ± 6.4% (n = 4; *P* < 0.05) reduction of viral RNA in human intestinal organoids (Fig. 1F). Thus, blocking PI3K by LY294002 potently inhibits rotavirus infection in both Caco2 cells and human intestinal organoids.

### mTOR sustains rotavirus infection and the mTOR inhibitor rapamycin inhibits its infection

**Figure 2.**
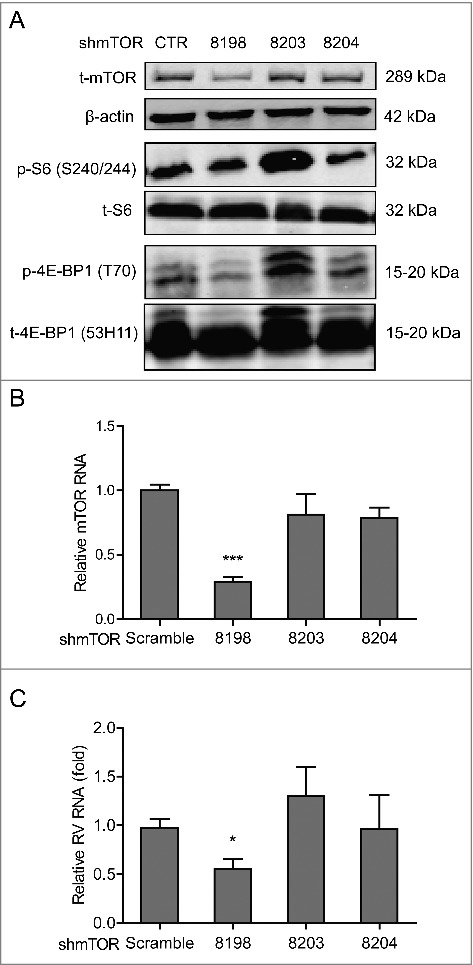
Silence of mTOR inhibits rotavirus replication. (A) Western blot analysis of t-mTOR, p-S6 (S240/244), t-S6, p-4E-BP1 (T70) and t-4E-BP1 (53H11) protein in lentiviral RNAi against mTOR transduced Caco2 cells. (B) One (No. 8198) of 3 lentiviral shRNA vectors showed successful knockdown determined by qRT-PCR (n = 9, mean ± SEM, ***P < 0.001, Mann-Whitney test). (C) mTOR knockdown inhibited rotavirus genomic RNA determined by qRT-PCR (n = 7, mean ± SEM, *P < 0.05, Mann-Whitney test).

**Figure 3.**
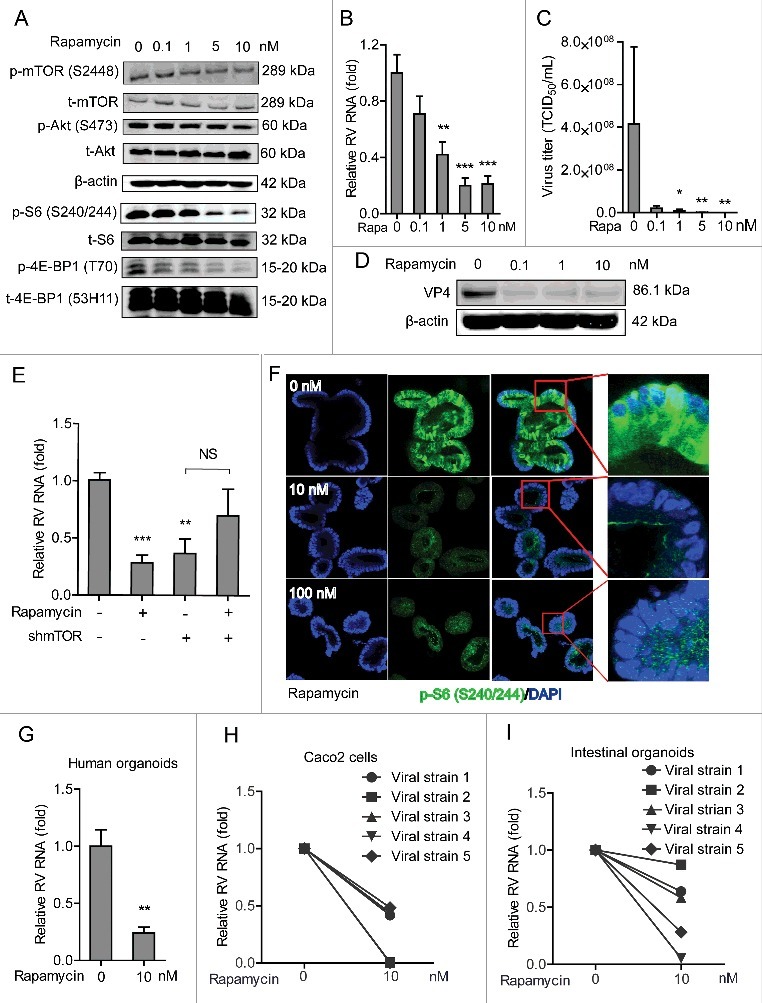
Rapamycin inhibits replication of SA11 and patient-derived rotavirus replication. (A) Western blot analysis of p-mTOR (S2448), t-mTOR, p-Akt (S473), t-Akt, p-S6 (S240/244), t-S6, p-4E-BP1 (T70) and t-4E-BP1 (53H11) protein levels with the treatment of indicated concentrations of rapamycin in Caco2 cells. (B) Treatment with rapamycin (48 h) significantly inhibited SA11 rotavirus replication in Caco2 cells (n = 7–9, mean ± SEM, **P < 0.01, Mann-Whitney test). (C) Effects of rapamycin on the production of infectious viral particles determined by TCID_50_ method. Each bar represents the TCID_50_/mL (mean ± SEM) (n = 5, *P < 0.05, **P < 0.01, Mann-Whitney test). (D) Treatment with rapamycin (48 h) inhibited viral VP4 protein determined by western blot assay in Caco2 cells. (E) Anti-rotavirus effect of rapamycin was abolished in mTOR knockdown Caco2 cells (n = 10, mean ± SEM, *P < 0.05, Mann-Whitney test). (F) Representative confocal immunostainings of p-S6 (S240/244) (Green) after 48 h treatment with 10 and 100 nM rapamycin in human intestinal organoids. Nuclei were visualized by DAPI (blue). (G) Treatment with rapamycin (48 h) inhibited SA11 rotavirus genomic RNA in human intestinal organoids determined by qRT-PCR (n = 5, mean ± SEM, *P < 0.01, Mann-Whitney test). (H) Treatment with 10 nM rapamycin (48 h) inhibited patient-derived rotavirus (isolate 1–5) genomic RNA in Caco2 cells determined by qRT-PCR. (I) Treatment with rapamycin (48 h) inhibited patient-derived rotavirus (isolate 1–5) genomic RNA in human intestinal organoids determined by qRT-PCR.

mTOR is a central element of the PI3K-Akt-mTOR signaling pathway, we thus investigated its effect on rotavirus infection. To address this, Caco2 cells were transduced with integrating lentiviral vectors expressing shRNA to silence mTOR. One (No 8198) out of 3 clones showed potent knockdown based on western blot results (Fig. 2A), which was also confirmed by qRT-PCR (Fig. 2B). Caco2 cells with mTOR knockdown or scrambled shRNA (as control) were inoculated with SA11 rotavirus and viral genomic RNA was quantified by qRT-PCR 48 h post inoculation. Silencing of mTOR led to a significant reduction of rotavirus RNA (Fig. 2C), suggesting a supportive role of mTOR for rotavirus infection. Rapamycin is a clinically used mTOR inhibitor, and it was approved by FDA to treat/prevent transplant allograft rejection.^25^ Phosphorylation of Ser-2448 mTOR, Ser-240/244 S6 and 4E-BP1 (T70) but not for the corresponding total proteins (as controls) were inhibited by treatment with 1, 5 and 10 nM rapamycin for 48 h in Caco2 cells (Fig. 3A and Figure S1B), except for phosphorylation of Ser-473 Akt. mTOR/S6K inhibition by rapamycin was reported to trigger a negative feedback loop to activate Akt signaling,^26^ which might be the reason that rapamycin had minor inhibitory effect on phosphorylated Akt. Furthermore, treatment of 5 and 10 nM rapamycin significantly inhibited viral genomic RNA by 80.0 ± 5.5% (n = 8; *P* < 0.01) and 79.9 ± 5.8% (n = 9; *P* < 0.01) in Caco2 cells, respectively (Fig. 3B). Rotavirus RNA secretion (Figure S2C) and infectious virus production (Fig. 3C) were also significantly inhibited by treatment of rapamycin. Consistently, western blot assay indicated that rotavirus VP4 protein synthesis was potently inhibited by rapamycin treatment (Fig. 3D). In contrast, rapamycin lost its anti-rotavirus activity in mTOR silenced Caco2 cells (Fig. 3E), supporting its specificity in inhibiting its biologic target to exert the antiviral action.

To verify the anti-rotavirus effect of rapamycin, human intestinal organoids infected with SA11 rotavirus were treated with 10 or 100 nM rapamycin for 48 h, which led to vigorous inhibition of phosphor-Ser-240/244 S6 (Fig. 3F). Importantly, 10 nM rapamycin resulted in a significant reduction (75.9 ± 5%, n = 5; *P* < 0.01) of viral RNA replication (Fig. 3G). This was further validated in 5 patient-derived strains by inoculation of Caco2 cells or human intestinal organoids with stool samples from rotavirus-infected patients (Table S2). Treatment with 10 nM rapamycin inhibited patient-derived isolates in both Caco2 cells (Fig. 3H) and human intestinal organoids (Fig. 3I). These results have demonstrated that efficient infection of rotavirus requires mTOR, but the infection can be effectively inhibited by rapamycin.

### Dual inhibition of PI3K and mTOR inhibits rotavirus infection

**Figure 4.**
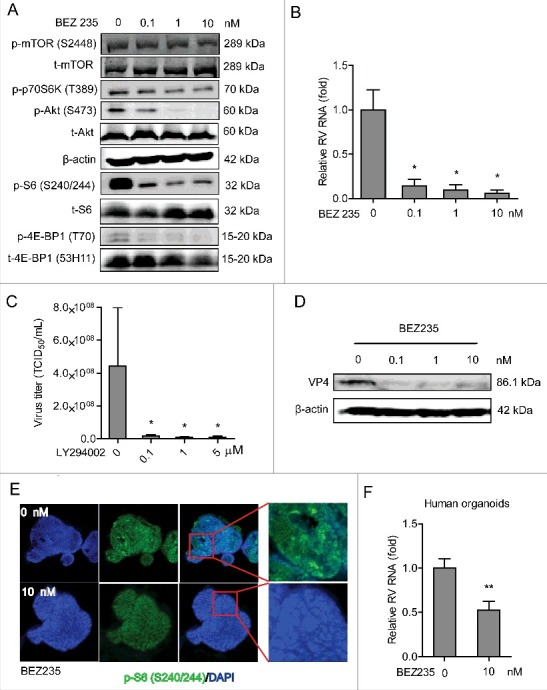
Dual inhibition of PI3K and mTOR inhibits rotavirus infection. (A) Western blot assay detected p-mTOR (S2448), p-p70S6K (T389), p-Akt (S473) and p-S6 (S240/244) and the corresponding total proteins after 48 h incubation with indicated concentrations of BEZ235 in Caco2 cells. (B) Treatment with BEZ235 (48 h) significantly inhibited SA11 rotavirus genomic RNA in dose-dependent-manner determined by qRT-PCR in Caco2 cells (n = 5, mean ± SEM, *P < 0.05, Mann-Whitney test). (C) Effects of BEZ235 on the production of infectious viral particles determined by TCID_50_ method. Each bar represents the TCID_50_/mL (mean ± SEM) (n = 5, *P < 0.05, **P < 0.01, Mann-Whitney test). (D) Western blot showed that treatment with BEZ235 (48 h) significantly inhibited SA11 rotavirus VP4 protein in Caco2 cells. (E) Representative confocal immunostainings of p-S6 (S240/244) (Green) after 48 h incubation with 0 (as a control) and 10 nM BEZ235 in human intestinal organoids. Nuclei were visualized by DAPI (blue). (F) Treatment with BEZ235 (48 h) significantly inhibited SA11 rotavirus genomic RNA in human intestinal organoids quantified by qRT-PCR (n = 11, mean ± SEM, **P < 0.01, Mann-Whitney test).

BEZ235 is a pharmacological dual inhibitor of PI3K and mTOR. Western blot confirmed that this compound could potently inhibit phosphorylation of Ser2448 mTOR, 70S6K (T389), Ser473 Akt, Ser240/244 S6 and 4E-BP1 (T70) but not for the corresponding total proteins (as controls) in Caco2 cells (Fig. 4A and Figure S1C). Importantly, BEZ235 dose-dependently inhibited viral RNA (Fig. 4B) and RNA secretion in Caco2 cells (Figure S2D). Using the TCID_50_ method, we have demonstrated that it remarkably inhibits the production of infectious viral particles (Fig. 4C). BEZ235 was also capable of inhibiting synthesis of rotavirus VP4 protein (Fig. 4D). This effect was also confirmed in human intestinal organoids of inhibiting phosphorylation of Ser240/244 S6 (Fig. 4E) and rotavirus infection (Fig. 4F). Although rotavirus infection did not directly affect the expression levels of the key components the PI3K-Akt-mTOR pathway (Figure S3), this pathway itself intrinsically sustained rotavirus infection.

### 4E-BP1, but not S6K, is a downstream effector of mTOR in sustaining rotavirus infection

**Figure 5.**
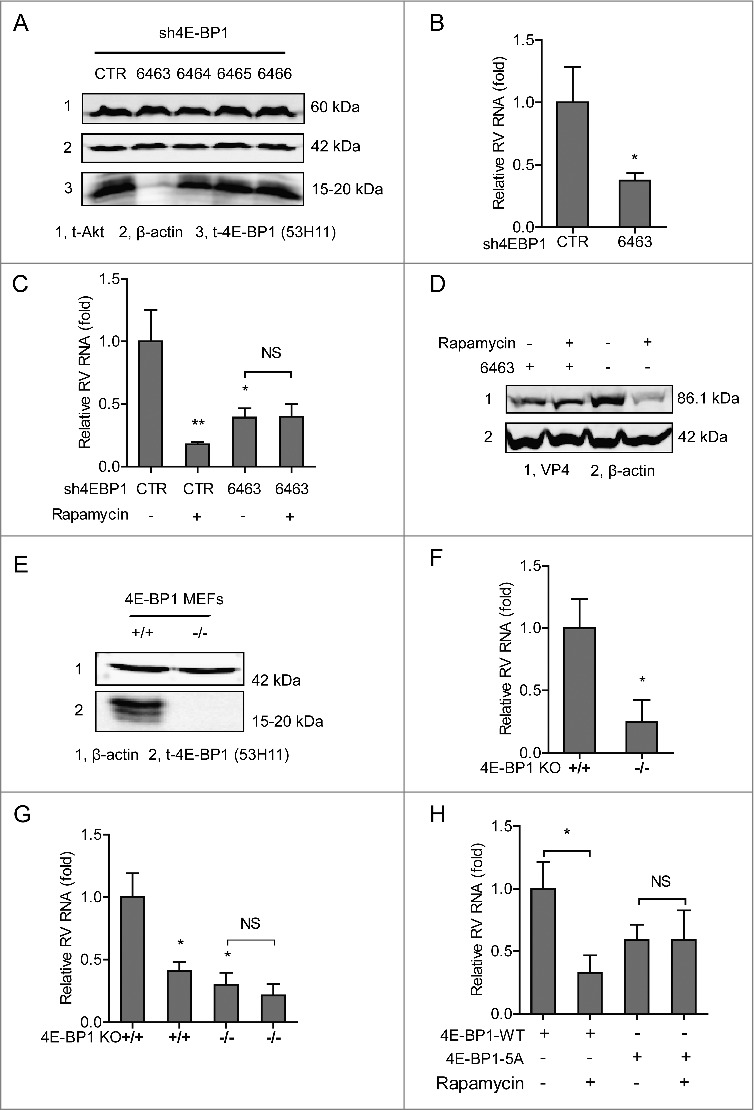
4E-BP1 is a downstream effector of PI3K-Akt-mTOR signaling in sustaining rotavirus infection. (A) Western blot assay detected t-Akt and t-4E-BP1 (53H11) in lentiviral RNAi against 4E-BP1 transduced Caco2 cells. (B) Knockdown of 4E-BP1 significantly inhibited SA11 rotavirus genomic RNA quantified by qRT-PCR (n = 5, mean ± SEM, *P < 0.05, Mann-Whitney test). (C) Anti-rotavirus effect of rapamycin (10 nM) was abolished in 4E-BP1 knockdown Caco2 cells (n = 5, mean ± SEM, *P < 0.05, **P < 0.01, Mann-Whitney test). (D) Western blot assay confirmed that anti-rotavirus effect of rapamycin (10 nM) was abolished in 4E-BP1 knockdown Caco2 cells. (E) Western blot indicated *bona fide* knockdown of 4E-BP1 in knockout (−/−) MEF cells. (F) SA11 rotavirus replication was potently restricted in 4E-BP1 knockout (−/−) MEF cells (n = 4, mean ± SEM, *P < 0.05, Mann-Whitney test). (G) Anti-rotavirus effect of rapamycin (10 nM) was attenuated in 4E-BP1 knockout (−/−) MEF cells (n = 8, mean ± SEM, *P < 0.05, Mann-Whitney test). (H) Rapamycin inhibited rotavirus replication in 4E-BP1 KO MEFs transfected with 4E-BP1-WT plasmids; while this antiviral effect was abolished in 4E-BP1 KO MEFs transfected with 4E-BP1–5A plasmids (n = 5, mean ± SEM, *P < 0.05, Mann-Whitney test).

4E-BP1 is one of the important downstream elements of PI3K-Akt-mTOR signaling. We first studied its potential involvement by lentiviral RNAi mediated loss-of-function assay (Fig. 5A and Figure S1D). Caco2 cells with the best knockdown of 4E-BP1 (no 6463) resulted in 62.4 ± 6.1% (n = 5, P <0.01) reduction of SA11 rotavirus viral RNA 48 h post-inoculation (Fig. 5B). Consistently, the anti-rotavirus effect of rapamycin was abolished in 4E-BP1 knockdown cells confirmed by both qRT-PCR assay (Fig. 5C) and western blot assay (Fig. 5D). To further verify, we obtained 4E-BP1 knockout mouse embryonic fibroblasts (MEFs), and *bona fide* knockout was confirmed by western blot assay (Fig. 5E). Consistently, rotavirus infection in 4E-BP1 knockout MEFs (−/−) is significantly less efficient (75.1 ± 17.2%; n = 4; *P* < 0.05), compared with the infection in wild type MEFs (+/+) (Fig. 5F). Furthermore, the anti-rotavirus effect of rapamycin was attenuated in 4E-BP1 knockout MEFs (Fig. 5G). Consistently, rapamycin could inhibit rotavirus replication in 4E-BP1 KO MEFs transfected with 4E-BP1 wild-type (4E-BP1-WT) plasmids; while this antiviral effect was abolished in 4E-BP1 KO MEFs transfected with 4E-BP1 plasmids having 5 mutations of the phosphorylation sites (4E-BP1–5A) (Fig. 5H). Of note, the inhibitor of S6K did not affect rotavirus infection (Figure S4). Collectively, these data suggest that 4E-BP1 but not S6K is a downstream effector of the PI3K-Akt-mTOR signaling in sustaining rotavirus infection.

### 4E-BP1 mediates rapamycin-induced autophagy that inhibits rotavirus infection

Previous studies have reported that rapamycin or 4E-BP1 may affect type I IFN signaling,^27-29^ in particular the downstream antiviral proteins including interferon regulatory factor 1 (IRF1) and IRF7. Furthermore, we previously have reported the anti-rotavirus effect of IFNα.^23^ We thus investigated whether these antiviral proteins may play a role in this setting. We found that rapamycin treatment did not affect the protein expression of IRF1 or IRF7 in Caco2 cells and MEFs (Figure S5A and B). Although the deficiency of 4E-BP1 appears to slightly increase IRF7 but not IRF1 protein synthesis (Figure S5B). Thus, these results indicate that these antiviral proteins are likely not essential in the anti-rotavirus action of the PI3K-Akt-mTOR signaling.

**Figure 6.**
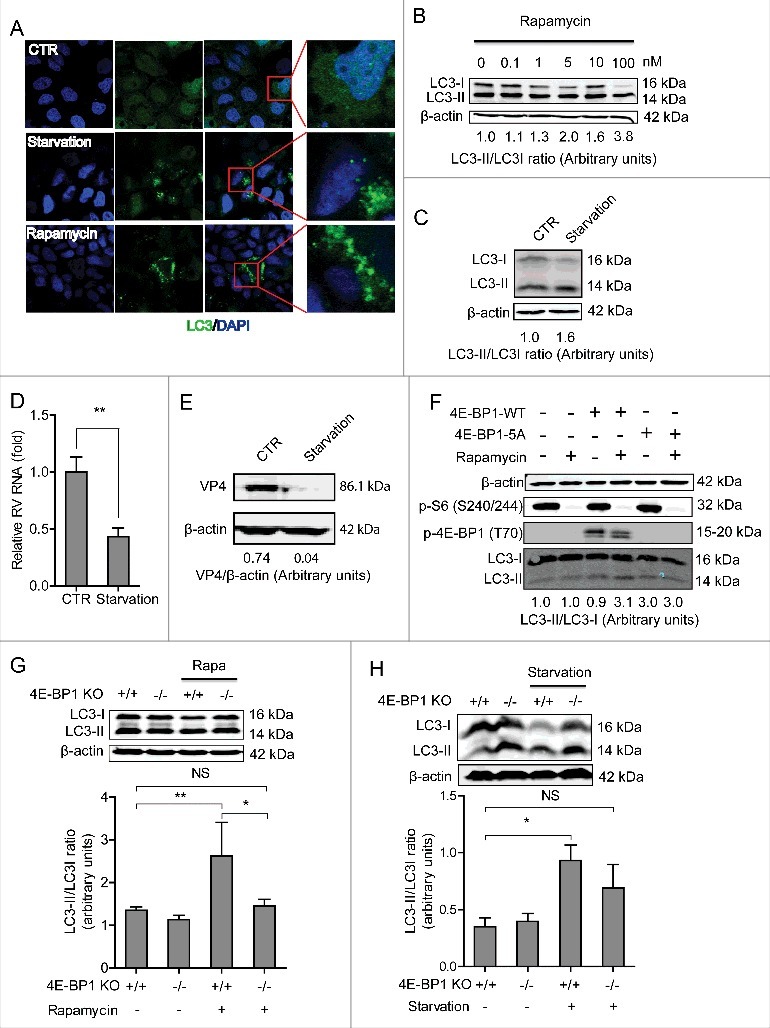
4E-BP1 mediates rapamycin-induced autophagy that inhibits rotavirus infection. (A) Caco2 cells transduced with lentiviral particles carrying a construct of TagGFP2-LC3 driven by the elongation factor-1 promotor were cultured at 37°C for 48 h in DMEM medium containing EBSS medium containing 1mM pepstatin A and E-64-d solution (for starvation) and 10 nM rapamycin. LC3-positive puncta was observed by confocal laser microscopy. (B) LC3-I and LC3-II protein levels were examined by western blot analysis. Protein samples were extracted from Caco2 cells treated with indicated concentrations of rapamycin (48 h). Quantification of the intensity of the immunoreactive bands of both LC3-I and LC3-II was performed using Odyssey V3.0 software. Densitometric analysis of immunoblots of LC3 was expressed as the ratio of LC3-II to LC3-I, and the ratio of LC3II/LC3I was expressed in arbitrary units. (C) Western blot visualized LC3-I and LC3-II protein levels in starvation (EBSS medium containing 1mM pepstatin A and E-64-d solution) treated Caco2 cells. The ratio of LC3II/LC3I was expressed in arbitrary units. (D) Starvation significantly inhibited rotavirus RNA in Caco2 cells (n = 6, mean ± SEM, *P < 0.05, Mann-Whitney test). (E) Starvation dramatically inhibited rotavirus protein VP4 synthesis in Caco2 cells. (F) Rapamycin induced autophagy in 4E-BP1 KO MEFs transfected with 4E-BP1-WT plasmids; while the induction was abolished in 4E-BP1 KO MEFs transfected with 4E-BP1–5A plasmids. (G) Western blot demonstrated that upregulated ratio of LC3-II to LC3-I by rapamycin was abolished in 4E-BP1 knockout (−/−) MEFs (n = 6, mean ± SEM, *P < 0.05, **P < 0.01, Mann-Whitney test). (H) Western blot detected that upregulated ratio of LC3-II to LC3-I by starvation (EBSS medium containing 1mM pepstatin A and E-64-d solution) was abolished in 4E-BP1 knockout (−/−) MEFs (n = 5, mean ± SEM, *P < 0.05, Mann-Whitney test).

We therefore redirected our attention to autophagy, a cellular metabolism process functioning in orderly degradation and recycling of cellular components, which has been implicated in regulating many types of virus infections.^30^ mTOR is the main inhibitor of autophagy. Rapamycin, as a specific inhibitor of mTOR, is a potent inducer of autophagy formation.^31^ We first confirmed that starvation (EBSS medium containing 1mM pepstatin A and E-64-d solution, as a positive control) stimulated the autophagy process in Caco2 cells (Fig. 6A). Consistently, the ratio of LC3-II to LC3-I (hallmark of autophagy induction) was upregulated by rapamycin treatment (Fig. 6B and Figure S1E) or treatment with starvation (Fig. 6C and Figure S1F). Similar to rapamycin treatment (Fig. 3B and D), starvation also significantly inhibited rotavirus mRNA (Fig. 6D) and viral protein synthesis (Fig. 6E and Figure S1G). Interestingly, induction of autophagy by rapamycin and starvation was confirmed to be via 4E-BP1. Because the autophagy is equally increased by rapamycin treatment, in which 4E-BP1 is not phosphorylated at all in the mutant (Fig. 6F). Consistently, the upregulation of LC3-II/LC3-I ratio by rapamycin or starvation was blocked in 4E-BP1 knockout MEFs cells (−/−) (Fig. 6G and H).

**Figure 7.**
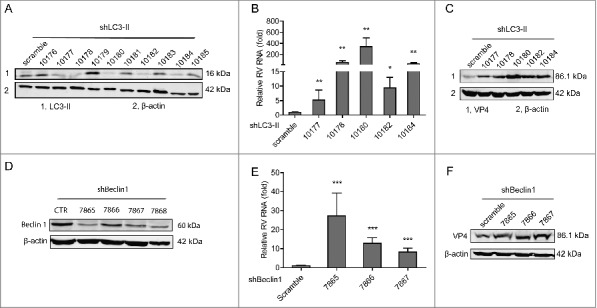
Silence of autophagy related genes, LC3-II and beclin1, inhibits rotavirus replication. (A) Western blot detected LC3-II protein in lentiviral RNAi against LC3-II transduced Caco2 cells. (B) LC3-II knockdown increased rotavirus genomic RNA determined by qRT-PCR (n = 4–10, mean ± SEM, *P < 0.05, **P < 0.01, Mann-Whitney test). (C) LC3-II knockdown increased rotavirus VP4 protein synthesis detected by western blot assay. (D) Western blot detected beclin1 protein in lentiviral RNAi against beclin1 transduced Caco2 cells. (E) Beclin1 knockdown increased rotavirus genomic RNA determined by qRT-PCR (n = 11–17, mean ± SEM, ***P < 0.001, Mann-Whitney test). (F) Beclin1 knockdown increased rotavirus VP4 protein synthesis detected by western blot assay.

To demonstrate the role of autophagy in rotavirus infection, we applied RNAi knockdown of key components of the autophagy machinery. To this aim, LC3 gene that is associated with the autophagosome membrane was silenced with 10 integrating lentiviral RNAi vectors. Five (no 10177, 10178, 10180, 10182 and 10184) out of them showed potent knockdown (Fig. 7A). Significant increase of rotavirus genomic RNA was found in all of the 5 knockdown Caco2 cell lines as quantified by qRT-PCR (Fig. 7B). It was further confirmed that viral protein synthesis was increased in these 5 knockdown Caco2 cell lines (Fig. 7C). Beclin1 plays an important role in localization of autophagic proteins to a pre-autophagosomal structure.^32^ Similarly, knockdown of Beclin1 also favored rotavirus infection (Fig. 7D, E and F). Of note, the 3 optimal knockdown (no 7865, 7866 and 7867) clones were found to increase rotavirus genomic RNA by 27.3 ± 11.9 (n = 11; *P* < 0.01), 12.8 ± 3.0 (n = 13; *P* < 0.001) and 8.2 ± 2.1 (n = 17; *P* < 0.001) fold, respectively (Fig. 7E). The viral protein synthesis was further confirmed to be increased in Beclin1 knockdown Caco2 cells (Fig. 7F). Thus, we conclude that 4E-BP1 mediates rapamycin-induced autophagy machinery in inhibiting rotavirus infection.

## Discussion

Many viruses interfere with or use PI3K-Akt-mTOR pathway to regulate their infection.^19^ Vesicular stomatitis virus (VSV) infection and replication have been reported to lead to dephosphorylation and inactivation of Akt.^33^ In contrast, influenza A virus replication activates Akt phosphorylation by the viral NS1 protein. It directly binds to the PI3K regulatory subunit (p85β), allowing the catalytic domain (p110) to phosphorylate Akt.^34^ Recent structural study has provided insights into the mechanism by which NS1 activates the PI3K (P85β:P110) holoenzyme.^35^ White spot syndrome virus (WSSV) stimulates phosphorylation of 4E-BP1, the downstream element of PI3K-Akt-mTOR pathway.^36^ Semliki Forest virus (SFV) and human papillomavirus type 16 (HPV16) have also been reported to phosphorylate the mTOR targets S6 kinase and 4E-BP1.^37,38^ In fact, PI3K-Akt-mTOR pathway serves as a pro- or antiviral double-edged sword depending on the types of viruses. The pathway itself has been demonstrated to exert inhibitory effect on hepatitis E virus (HEV) replication via 4E-BP1.^13^ In contrast, it is also involved in promoting the infection of a broad-spectrum of viruses. Thus, blocking PI3K by its inhibitor could vigorously inhibit infection of numerous viruses including LCMV^19^ and coxsackievirus.^39^ Over-expression of p70S6K or Akt stimulates mRNA expression of coxsackievirus B3 (CVB3).^40^ Rotavirus infection has been reported to activate phosphorylation of p70S6K in piglets.^41^ However, we did not found evidence that rotavirus infection induced phosphorylation of 4E-BP1, S6, Akt or p70S6K in our system (cells of human origin). The underlying discrepancy might derive from the differences of the species and distinct physiologic situation, since animal models in some cases are of limited relevance to human physiology.^42^ However, this pathway is essential in sustaining rotavirus infection, and inhibition of PI3K-Akt-mTOR signaling resulted in potent anti-rotavirus activity (Figs. 1, 2, 3, 4 and 5).

PI3K-Akt-mTOR pathway functions by phosphorylation of its downstream effectors including ribosomal protein S6 Kinase 1 (S6K1) and 4E-BP1 to upregulate cap-dependent mRNA translation.^43^ Merkel cell polyomavirus (MCV) stirs phosphorylation of 4E-BP1. Subsequently, it disassociates from eIF4E to form the eIF4F complex to induce mRNA translation.^44^ Rift valley fever virus (RVFV) induces decay of 5′-TOP mRNAs via 4E-BP1 to restrict virus replication.^45^ We have demonstrated that the anti-rotavirus effect by inhibition of PI3K-Akt-mTOR pathway is predominantly via 4E-BP1 but not S6K. It has been reported that 4E-BP1 mediates the inhibitory effect of mTOR on autophagy formation.^46^ In this study, we confirmed that autophagy was induced by inhibiting mTOR with treatment of rapamycin in our system, and this process is mediated by 4E-BP1.

The process of autophagy induction has been implicated in regulating many types of virus infection with various mechanism-of-action.^31,47^ We have functionally demonstrated that autophagy machinery exerts antiviral effect against rotavirus (Fig. 6D and E). Some viruses including human cytomegalovirus,^48^ hepatitis B virus-X (HBx) ^49^ and chikungunya virus ^50^ have been reported to lure autophagy machinery in host. Rotavirus was also reported to induce autophagy in piglet intestine.^51^ However, we did not observe changed ratio of LC3-II to LC3-I upon rotavirus infection. The key autophagy related genes have been well studied in regulating virus infections with different model-of-actions. During Sindbis virus infection, beclin 1 expression was upregulated that may result in xenophagy (a selective autophagy) of Sindbis virus to defend the infection.^52^ Several autophagy related genes can promote both innate and adaptive immune response to combat viral infection.^53^ We demonstrated that silence of LC3-II and beclin1 led to potent increase of rotavirus infection (Fig. 7).

In the clinic, organ transplantation patients irrespective to their age are very sensitive to rotavirus infection. Long-term use of immunosuppressants is an important risk factor causing persistent infection in these patients.^4^ Rapamycin as a potent mTOR inhibitor has been used as immunosuppressant in transplant patients for decades.^54^ It has been well documented that different types of immunosuppressants may differentially affect virus infection.^4,55^ Antiviral effects of rapamycin have been demonstrated in models of herpes simplex virus,^56^ coxsackievirus^39^ and HIV-1 infections.^21^ Our data demonstrating potent anti-rotavirus effect of rapamycin favor the clinical application of this immunosuppressant in rotavirus-infected organ recipients.

**Figure 8.**
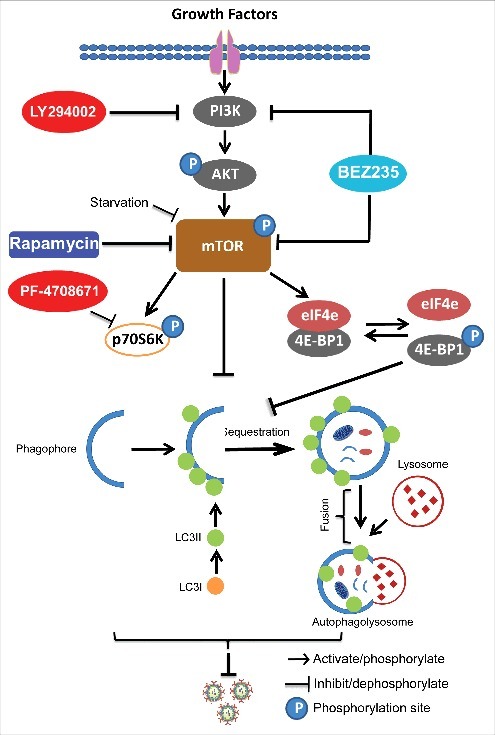
Schematic depicting the PI3K-Akt-mTOR signaling involved in regulating rotavirus replication. Pharmaceutical blocking PI3K-Akt-mTOR pathway inhibits phosphorylation and activation of its downstream targets including p70S6K and 4E-BP1. 4E-BP1 mediates inhibition of mTOR on autophagy machinery via 4E-BP1. Autophagy itself exerts anti-rotavirus effect.

In summary (Fig. 8), we have dissected the effects of PI3K-Akt-mTOR signaling on rotavirus infection, and demonstrated that blocking this pathway can potently inhibit rotavirus replication. Importantly, the mTOR inhibitor, rapamycin, induced autophagy machinery to inhibit rotavirus infection via 4E-BP1. Thus, this study revealed new insights on rotavirus-host interactions and provided new avenues for antiviral drug development. Furthermore, it provides important references for transplant clinicians to design optimal immunosuppression protocols for rotavirus infected organ transplant patients.

## Materials and methods

### Reagents

Stocks of LY294002, an inhibitor of PI3K-Akt (Sigma-Aldrich), BEZ235, a dual inhibitor of PI3K-Akt and mTOR (Selleck Chemicals), rapamycin, a mTOR inhibitor (Merk, Schiphol-Rijk, The Netherlands) and PF-4708671, an inhibitor of p70S6K (Selleck Chemicals) were dissolved in dimethyl sulfoxide (DMSO) (Sigma-Aldrich, St Louis, MO). All reagents were stored in 25 μL aliquots and frozen at −80°C. Other reagents including Earle's Balanced Salt Solution (EBSS) (Lonza), E-64-d (Santa Cruz Biotech, Santa Cruz, CA) and pepstatin A (Santa Cruz Biotech, Santa Cruz, CA) were also used.

### Viruses

Simian rotavirus SA11 was used and prepared as described previously.^23^ Patient-derived rotavirus isolates were isolated from stool samples of rotavirus-infected patients during diarrhea period as described previously.^23^

### Cell and human primary intestinal organoid culture

Caco2 (Caucasian colon adenocarcinoma) cells are the heterogeneous human epithelial colorectal adenocarcinoma cells, representing numerous characteristics of enterocytes. They are commonly used for *in vitro* modeling rotavirus infection. Caco2 cells were maintained in culture in T25/T75 flask with Dulbecco's modified Eagle medium (DMEM) (Invitrogen-Gibco, Breda, The Netherlands) as described previously.^23^ Immortalized mouse embryonic fibroblasts (MEFs) derived from 4E-BP1 wild-type (+/+) and 4E-BP1 knockout (−/−) mice (gifts from E.N. Fish's laboratory) were cultured in DMEM (Invitrogen-Gibco, Breda, The Netherlands) containing 10% vol/vol fetal calf serum (FCS) (Hyclone, Lonan, Utah), 100 IU/mL penicillin, 100 mg/mL streptomycin and 2 mM L-glutamine (Invitrogen-Gibco).

3D cultures of human intestinal organoids were performed as described previously.^23^ Briefly, intestinal tissues were vigorously shaken in 8 mM EDTA for 15 min at 4°C, followed by removing the EDTA solution. Subsequently loosened crypts were collected by pipetting the solution up and down through a 10 mL pipette for 8–10 times. The crypt suspension was transferred into a 50 mL centrifuge tube (Greiner bio-one, the Netherlands) and biopsies were re-used for repeated collection of crypts (2–3 times). Crypt suspensions were pooled and centrifuged at 300 g for 5 min. Pelleted crypts were re-suspended in 2 mL complete medium with growth factors CMGF-: advanced DMEM/F12 supplemented with 1% (vol/vol) GlutaMAX™ Supplement (Gibco, Grand island, USA), 10 mM HEPES, and collected by centrifugation at 130 g for 5 min at 4°C. Crypts were finally suspended in Matrigel (Corning, Bedford, USA), and placed in the center of a well of a 24-well plate (40 µL per well). After the Matrigel had solidified (15 min at 37°C), organoids were cultured in culture medium at 37°C, 5% CO_2_. Culture medium was refreshed every 2–3 days, and organoids were passaged every 6–7 d.

### TCID_50_ assay

Reed and Muench method was applied to determine infectious virus titration with a standard TCID_50_ protocol by means of MA104 cells.^57^

### Virus infections and treatment of drugs

The protocols of inoculation of Caco2 cells with SA11 and patient-derived rotavirus were described previously.^23^ Briefly, cell monolayers of Caco2 cells were incubated with SA11 rotavirus at 37°C with 5% CO_2_ for 60 min for infection, followed by removing free virus particles. Subsequently, cells were added with culture medium (FCS free) containing 5 µg/mL of trypsin and indicative drugs, followed by incubation at 37°C in a humidified 5% CO_2_ incubator.

Organoids were inoculated with activated virus (5,000 genome copies) for 60 min at 37°C with 5% CO_2_, followed by removing free viral particles. Then, organoids were aliquoted in wells of a 48-well plate coated with 20% (vol/vol) Collagen R Solution (SERVA, Heidelberg, Germany) and culture medium containing drugs of interest was added. Subsequently, the 48-well plate containing organoids was spin down at 500 g for 5 minutes to promote the adherence on the bottom of the wells, followed by maintaining them at 37°C with 5% CO_2_.

Preparation of patient-derived rotavirus was performed as described earlier,^23^ and protocols of viral inoculation and treatment of drugs are similar to SA11 rotavirus. 48 h post-infection was used to determine viral replication levels, since it is the optimal time point for viral replication without lysis of the cells.

### RNA isolation, cDNA synthesis and qRT-PCR

Total RNA was extracted using a NucleoSpin® RNA kit (MACHEREY-NAGEL, Düren, Germany) and quantified with a Nanodrop ND-1000 (Wilmington, DE, USA). 500 ng of RNA was used as template for cDNA preparation with the reverse transcription system from TAKARA (TAKARA BIO INC). Quantitative real-time PCR (qRT-PCR) reactions were performed with SYBR Green (Applied Biosystems®, Austin, USA) according to the manufacturer's instruction. qRT-PCR was performed in an IQ5 cycler PCR machine (Bio-Rad). The levels of glyceraldehyde 3-phosphate dehydrogenase (GAPDH) mRNA were used as an endogenous reference to normalize the quantities of target mRNA by using the formula 2^−ΔΔCT^ (ΔΔCT = ΔCT_sample_-ΔCT_control_). All primer sequences were indicated in Table S1.

### Gene silencing assays

Lentiviral pLKO knockdown vectors (Sigma-Aldrich) targeting mTOR, 4E-BP1 and non-targeted control lentivirus were obtained from the Erasmus Biomics Center and produced in human embryonic kidney cell-line 293T (HEK293T) cells.

Caco2 cells were transduced with lentiviral vectors to generate stable gene knockdown cells. Transduced cells were subsequently selected with 6 μg/mL puromycin (Sigma). After pilot study, the shRNA vectors exerting optimal gene knockdown were selected. Knockdown and control Caco2 cells were incubated with rotavirus as described in the foregoing.

### Transient transfection

The plasmids including 4E-BP1-WT and 4E-BP1 5A were transfected in 4E-BP1 knockout MEFs. Cells were seeded in 6-well plates till ∼70% confluence, followed by adding 200 µL of Opti-MEM® reduced serum medium (Thermo Fisher Scientific) with 2 µg 4E-BP1-WT and 4E-BP1–5A plasmids and 6 µL Fu-GENE 6 transfection reagent (Roche Applied Science, Inc.). Transfected cells were incubated in complete medium for 24 h, followed by refreshing with 100 nM rapamycin or control medium for 48 h. The cells were analyzed by western blot after treatment. The experiments were executed in triplicate.

### Cell lysis, SDS-PAGE and western blotting

After treatment and washing with PBS, cells in 6-well plates were lysed with 400 μL of laemmli buffer (20% [vol/vol] glycerol, 4% [wt/vol] SDS and 120 mM Tris-HCl, PH 6.8) containing 100 mM DTT, and boiled for 10 min at 95°C. Then, cell lysates were subjected to SDS-PAGE, and proteins were transferred to a polyvinylidene difluoride (PVDF) membrane (Immobilon-FL). Subsequently, the membrane was blocked with 2.5 mL blocking buffer and 2.5 mL PBS containing 0.05% Tween 20 (PBS-T) for 1 hour at room temperature. It was followed by incubation with P-mTOR (1:1000, rabbit; cell signaling), t-mTOR (1:1000, rabbit; cell signaling), p-Akt (1:1000, rabbit; cell signaling), t-Akt (1:1000, rabbit; cell signaling), p-S6 (1:1000, rabbit; cell signaling), t-S6 (1:1000, rabbit; cell signaling), p-4E-BP1 (1:1000, rabbit; cell signaling), t-4E-BP1 (1:1000, rabbit; cell signaling), LC3-I/II (1:1000, rabbit; cell signaling) and SA11 rotavirus VP4 (1:1000, HS-2, mouse monoclonal (provided by professor Harry Greenberg, Stanford University School of Medicine, USA) overnight in 5 mL of blocking buffer/PBST (1:1) cocktail at 4°C. After 3 more washes with PBST, the immunoreactive bands were detected by western blot analysis and detection of β-actin served as loading control (1:1000, mouse monoclonal; Santa Cruz).

### Cytospin preparations and Confocal Laser Scanning Microscopy (CLSM) for organoids

Organoids were harvested from Matrigel using cold PBS, followed by fixing in 4% paraformaldehyde in PBS at 4°C for 10 min. Fixed organoids were added into the appropriate wells of the CytoSpin II Cytocentrifuge (Shandon Scientifi Ltd, Runcorn, England), and spin down at 1000 rpm for 2 min. The slides containing organoids were rinsed in PBS-baths 3 × 5 min, followed by treatment with 0.1% (vol/vol) Triton-x100 for 4 min. Then, the slides were rinsed in PBS-baths 2 × 5 min, followed by being incubated with milk-tween-glycine medium (0.05% tween, 0.5% skim milk and 0.15% glycine) to block background staining for 30 min. The slides were incubated in a humidity chamber with p-S6 antibodies (1:100, rabbit; cell signaling) diluted in milk-tween-glycine medium at 4°C overnight. The slides were washed 3 × 5 min in PBS-baths before 1 h incubation with 1:1000 dilutions of the anti-rabbit IgG (H^+^L), F(ab')2 Fragment (Alexa Fluor® 488 Conjugate) secondary antibody. Nuclei were stained with DAPI (4, 6-diamidino-2-phenylindole; Invitrogen). Images were detected using confocal electroscope.

### Lentiviral expression of green fluorescence protein bound to LC3 and live imaging

Caco2 cells were cultured on cover slide on the bottom of wells of a 6-well-plate with culture medium and transduced with lentiviral particles carrying a construct of TagGFP2-LC3 driven by the elongation factor-1 promoter (Millipore LentiBrite) at a multiplicity of infection (MOI) of 50 for 48 h. Then, the medium was changed into the pharmacological treatment of indicated time period. Live images were obtained by using confocal electroscope.

### Cytotoxicity assays in cells and organoids

The effects of chemical reagents on cell viability were determined by MTT assay (Figure S6). Briefly, Caco2 cells were seeded 1 × 10^4^ cells per well of a 96-well plate and viable cells were detected at indicated time points by adding 10 μL 5 mg/mL MTT per well, 3 h incubation at 37°C and replacement of the medium by 100 μL DMSO (Sigma). Absorbance (490 nm) was analyzed.

Intestinal organoids were embedded in Matrigel and cultured in wells of 24-well-plate with organoids culture medium containing relevant chemical reagents. The effects of related chemical reagents on intestinal organoids viability were determined by morphology of organoids observed by light microscope (Figure S7).

### Statistical analyses

All numerical results are expressed as Mean ± SEM. Statistical comparisons were analyzed by Mann-Whitney test for the data without normal distribution or t test for the data with normal distribution. P-values less than 0.05 were considered to be statistically significant. Analysis was performed using GraphPad Prism Version 5 (GraphPad Software Inc., La Jolla, CA).

## Supplementary Material

KVIR_S_1326443.zip

## Abbreviations

4E-BP1: 4E-binding protein 1
Akt: protein kinase B
DMEM: Dulbecco's modified Eagle medium
DMSO: dimethyl sulfoxide
FCS: fetal calf serum
HBx: hepatitis B virus-X
IRF1: interferon regulatory factor 1
LCMV: lymphocytic choriomeningitis virus
MCV: merkel cell polyomavirus
MEFs: mouse embryonic fibroblasts
MERS-CoV: Middle East Respiratory Syndrome Coronavirus
MOI: multiplicity of infection
mTOR: mammalian target of rapamycin
p70S6K: p70S6 kinase
PI3K: phosphatidylinositol 3-kinase
RVFV: rift valley fever virus
SFV: semliki forest virus; HEV, hepatitis E virus
WSSV: white spot syndrome virus
