# Supplementary material for: PI3K-Akt-mTOR axis sustains rotavirus infection via the 4E-BP1 mediated autophagy pathway and represents an antiviral target
Source: Virulence. 2017 Jun 1;9(1):83–98. doi: 10.1080/21505594.2017.1326443 (PMC5955461; doi:10.1080/21505594.2017.1326443)
Supplement: KVIR_S_1326443.zip [file kvir-09-01-1326443-s001.zip › KVIR_S_1326443.docx]

**Supplementary Tables & Figures**

**PI3K-Akt-mTOR axis sustains rotavirus infection via the 4E-BP1 mediated autophagy pathway and represents an antiviral target**

Yuebang Yin,^1^ Wen Dang,^1^ Xinying Zhou,^1^ Lei Xu,^1^ Wenshi Wang,^1^ Wanlu Cao,^1^ Sunrui Chen,^1^ Junhong Su,^2^ Xuepeng Cai,^3^ Shaobo Xiao,^4^ Maikel P. Peppelenbosch,^1^ and Qiuwei Pan^1*^

^1^Department of Gastroenterology and Hepatology, Erasmus MC-University Medical Center, Rotterdam, The Netherlands.

^2^Medical Faculty, Kunming University of Science and Technology, Kunming, PR China

^3^State Key Laboratory of Veterinary Etiological Biology, Lanzhou Veterinary Research Institute, Chinese Academy of Agricultural Sciences (CAAS), Lanzhou 730030, PR China.

^4^State Key Laboratory of Agricultural Microbiology, College of Veterinary Medicine, Huazhong Agricultural University, Wuhan 430070, China

^∗^To whom correspondence should be addressed:

Qiuwei Pan, Department of Gastroenterology and Hepatology, Erasmus MC, room Na-617, 'sGravendijkwal 230, NL-3015 CE Rotterdam, The Netherlands, Phone: +31(0)107037502, Fax: +31-(0)107032793. E-mail: q.pan@erasmusmc.nl

Table S1. Primers used in the study

Sequence of rotavirus primers

|  | SA11 Rotavirus | Human Patient Rotavirus |
| --- | --- | --- |
| Sense | TGGTTAAACGCAGGATCGGA | ACCATCTACACATGACCCTC |
| Anti-sense | AACCTTTCCGCGTCTGGTAG | CACATAACGCCCCTATAGCC |

Primer sequences of other genes

| Primers | Human | Mouse |
| --- | --- | --- |
| GAPDH-F | GTCTCCTCTGACTTCAACAGCG | TTCCAGTATGACTCCACTCACGG |
| GAPDH-R | ACCACCCTGTTGCTGTAGTAGCCAA | TGAAGACACCAGTAGACTCCACGAC |
| mTOR-F | AGCATCGGATGCTTAGGAGTGG |  |
| mTOR-R | CAGCCAGTCATCTTTGGAGACC |  |
| 4E-BP1-F | CACCAGCCCTTCCAGTGATGAG |  |
| 4E-BP1-R | CCTTGGTAGTGCTCCACACGAT |  |

Table S2. Patient characteristics.

| Patient | Age (yrs) | Gender | Symptoms | Virus Detection | | | | | | | |
| --- | --- | --- | --- | --- | --- | --- | --- | --- | --- | --- | --- |
|  |  |  |  | Enterovirus | Parechovirus | Norovirus genegroups I | Norovirus genegroups II | Adenovirus | Astrovirus | Sapovirus | Rotavirus |
| 1 | 3.5 | Female | Fever | No | No | No | No | No | No | No | Yes |
| 2 | 74 | Female | Congestive heart failure, myocarditis | No | No | No | No | No | No | No | Yes |
| 3 | 27 | Female | Fever, diarrhea, nausea, vomiting | No | No | No | No | No | No | No | Yes |
| 4 | 67 | Male | Fever, stomach ache, watery diarrhea (Kidney transplant) | No | No | No | No | No | No | No | Yes |
| 5 | 28 | Female | Nausea, stomach ache, watery diarrhea, fever, headache, vomiting | No | No | No | No | Yes | No | No | Yes |

**Supplementary Figures**


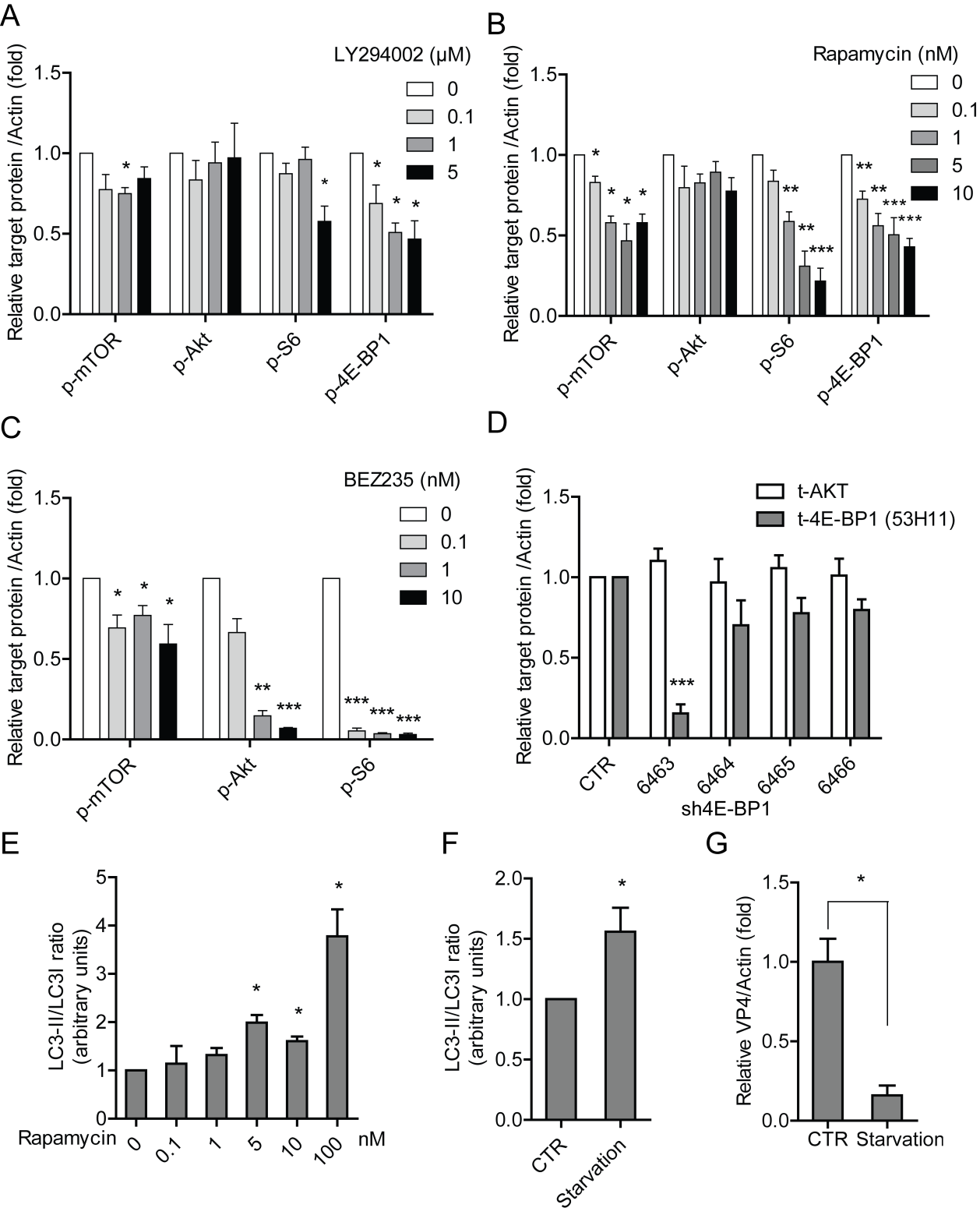


**Figure S1** Quantification of the intensity of the immunoreactive bands of p-mTOR, p-Akt, p-S6 and p-4E-BP1 with treatments of (A) LY294002 (n = 4, mean ± SEM, **P*< 0.05, t test), (B) rapamycin (n = 4-7, mean ± SEM, **P* < 0.05, ***P* < 0.01, ****P* < 0.001, t test) or (C) BEZ235 (n = 4, mean ± SEM, **P* < 0.05, ***P* < 0.01, ****P* < 0.001, t test) at indicated concentrations using Odyssey V3.0 software. (D) Quantification of the intensity of the immunoreactive bands of t-Akt and t-4E-BP1 (53H11) in 4E-BP1 knockdown Caco2 cells using Odyssey V3.0 software (n = 4, mean ± SEM, **P*< 0.001, t test) (E) Effect of rapamycin on autophagy (n = 3, means ± SEM, *P < 0.05, t test). Quantification of the intensity of the immunoreactive bands of both LC3-I and LC3-II was carried out using Odyssey V3.0 software. Densitometric analysis of immunoblots of LC3 was expressed as the ratio of LC3-II to LC3-I, and the ratio of LC3II/LC3I was expressed in arbitrary units. (F) Effect of starvation on autophagy (n = 3, means ± SEM, *P < 0.05, t test). Quantification of the intensity of the immunoreactive bands of both LC3-I and LC3-II was carried out using Odyssey V3.0 software. The ratio of LC3II/LC3I was expressed in arbitrary units. (G) Quantification of the intensity of the immunoreactive bands of VP4 with treatment of starvation using Odyssey. Data were presented as means ± SEM, *P< 0.05, **P< 0.01, **P< 0.001.


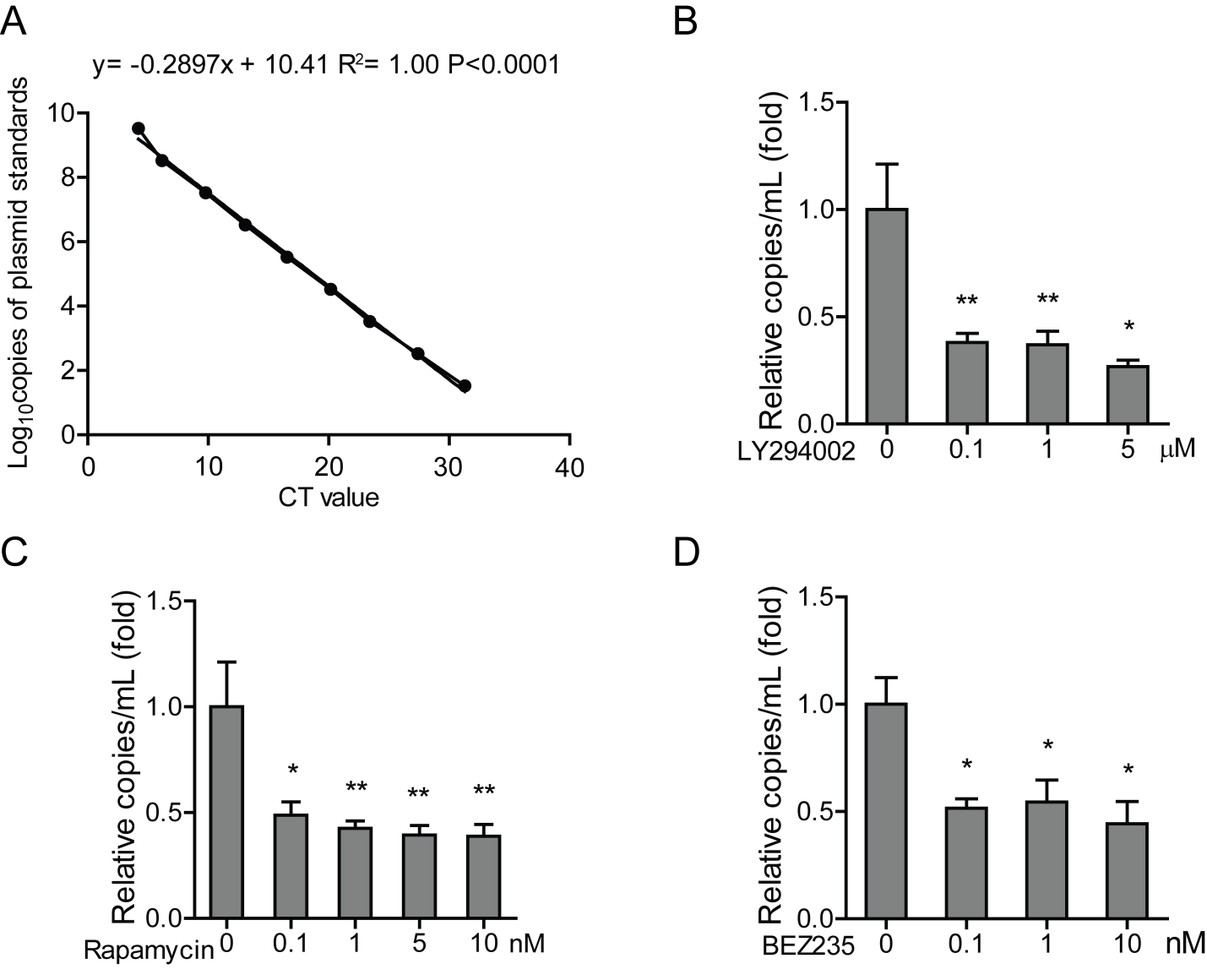


**Figure S2** Effects of (B) LY294002 (n = 6), (C) rapamycin (n = 6) and (D) BEZ235 (n = 5) on rotavirus secretion calculated by (A) a standard curves described previously (1). Caco2 cell monolayers were infected by trypsin activated rotavirus for 1 h, followed by removing free viral particles by washing with PBS for four times. Then, culture medium with indicative drugs was added for 48 h incubation, followed by that secreted viral particles in the medium were detected by qRT-PCR. Data were presented as means ± SEM, *P< 0.05, **P< 0.01.


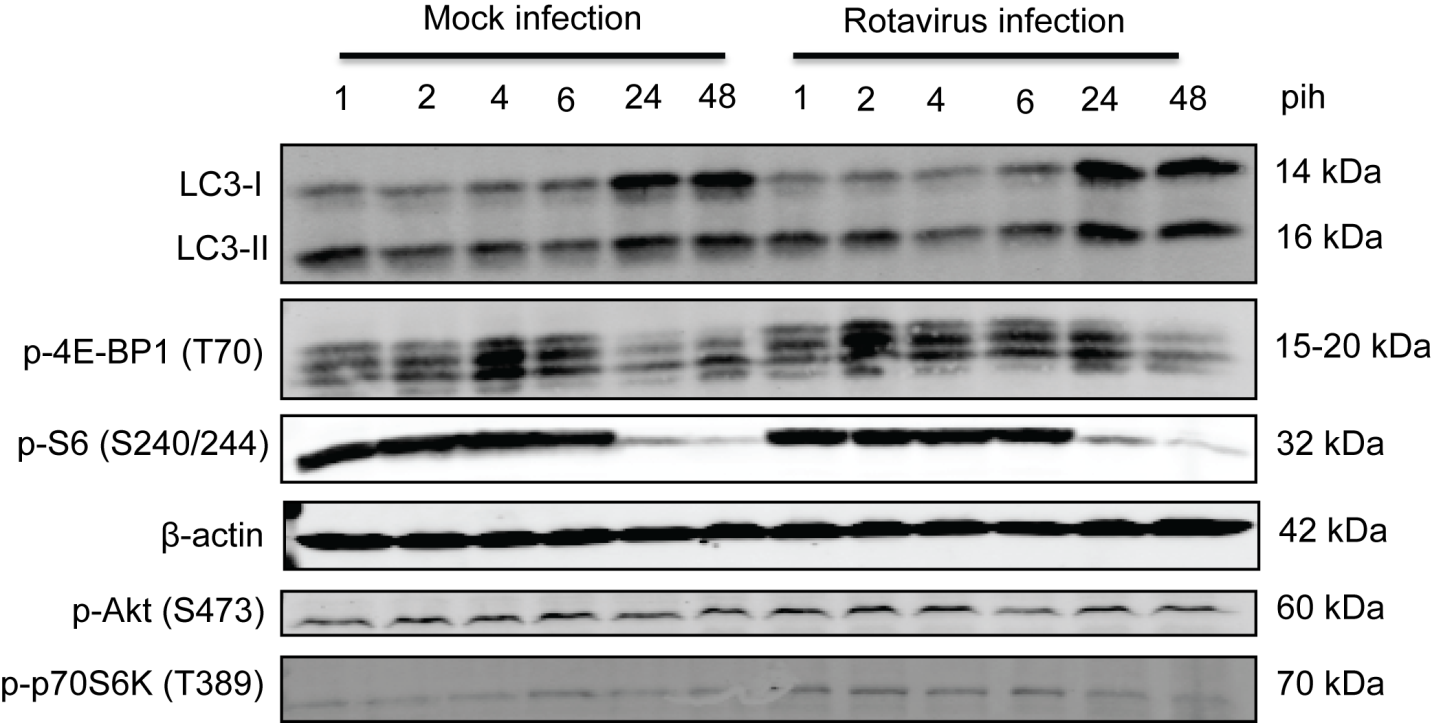


Figure S3 Rotavirus infection did not affect the key elements of PI3K-Akt-mTOR pathway. Western blot detects phosphorylated 4E-BP1 (T70), S6 (S240/244), Akt (S473), p70S6K (T389) and LC3-I/II proteins in mock and rotavirus infected Caco2 cells at indicated time points.


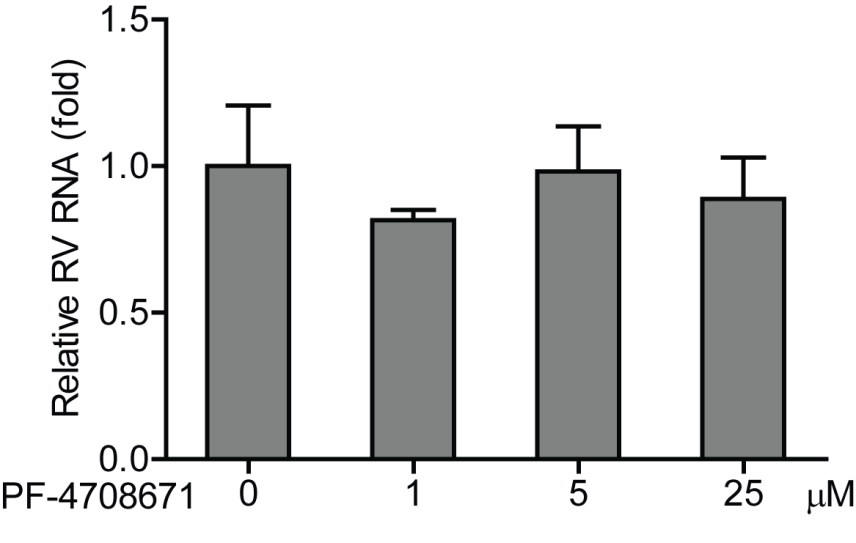


Figure S4 Effect of PF-4708671 (p70 ribosomal S6 kinase inhibitor) on rotavirus replication in Caco2 cells. Rotavirus infected Caco2 cells were treated with 1, 5 and 25 μM PF-4708671 for 48 h. Viral genomic RNA was quantified by qRT-PCR.


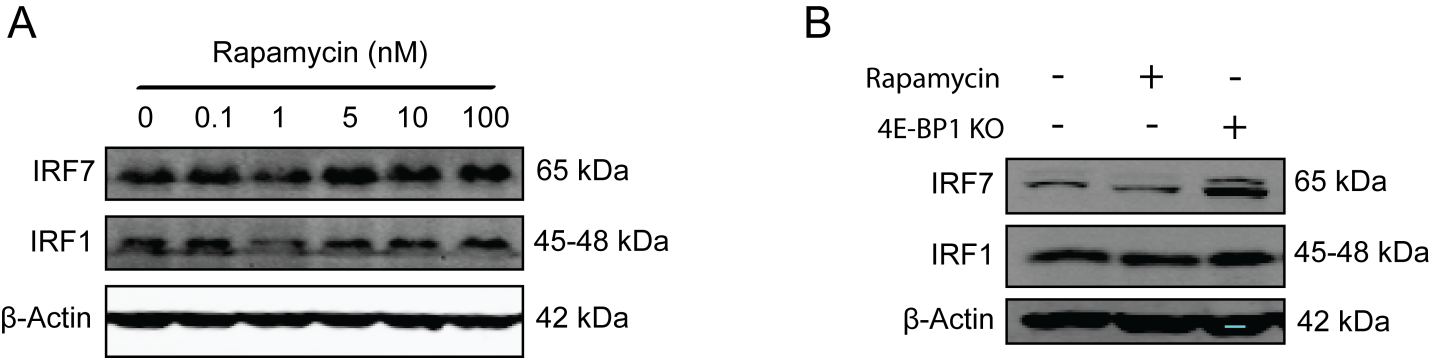


**Figure S5** Effects of rapamycin (A) and 4E-BP1 knock out (B) on IRF1 and IRF7 protein systhesis detected by western blot assay.


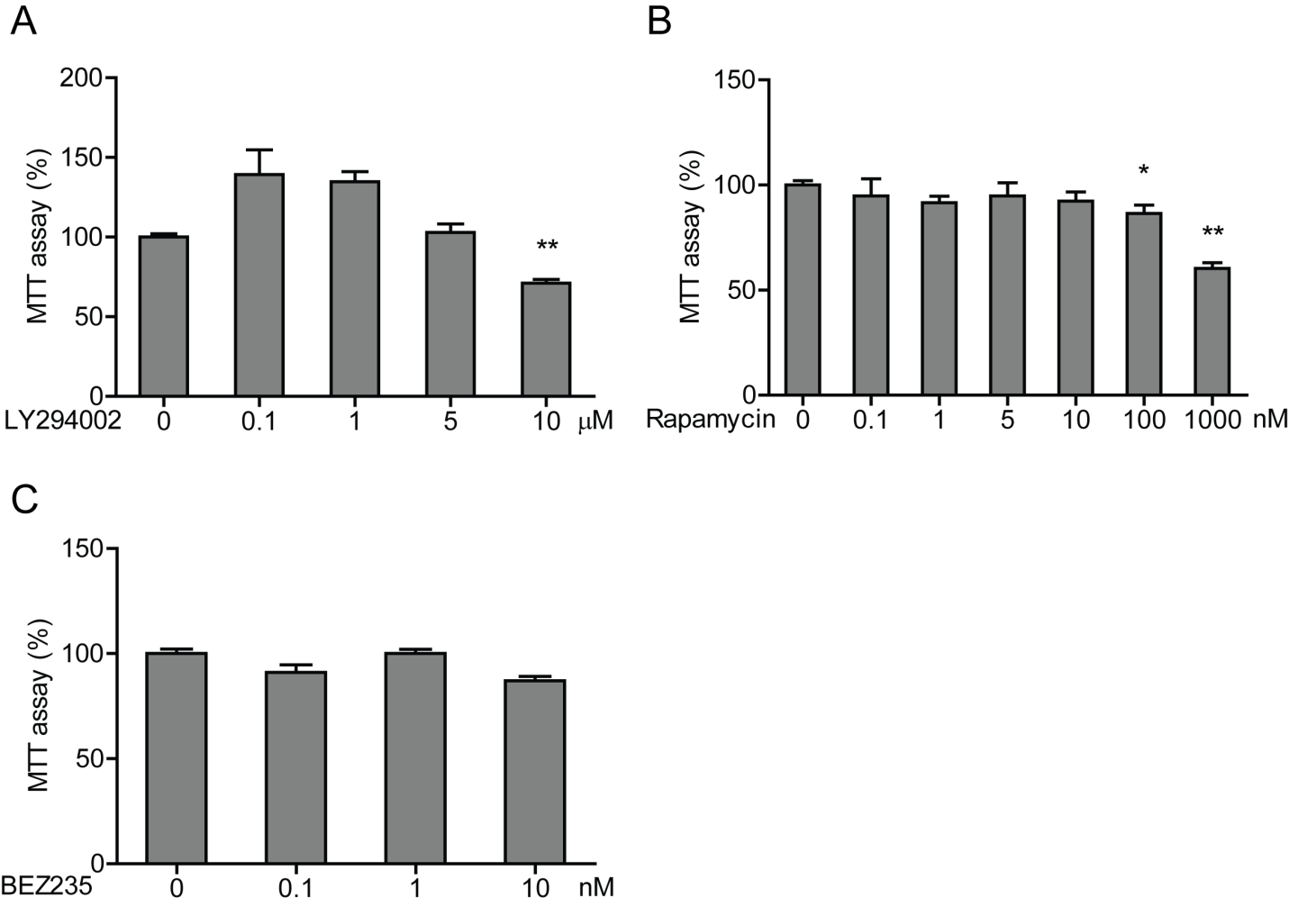


Figure S6 Effects of LY294002, BEZ235 and rapamycin on host cell viability determined by MTT assays. (A) Effect of LY294002 on viability of Caco2 cells (48 h) (n = 6, means ± SEM, **P< 0.01, Mann-Whitney test). (B) Effect of rapamycin on viability of Caco2 cells (48 h) (n = 6, means ± SEM, *P< 0.05, **P< 0.01, Mann-Whitney test). (C) Effect of BEZ235 on viability of Caco2 cells (48 h).


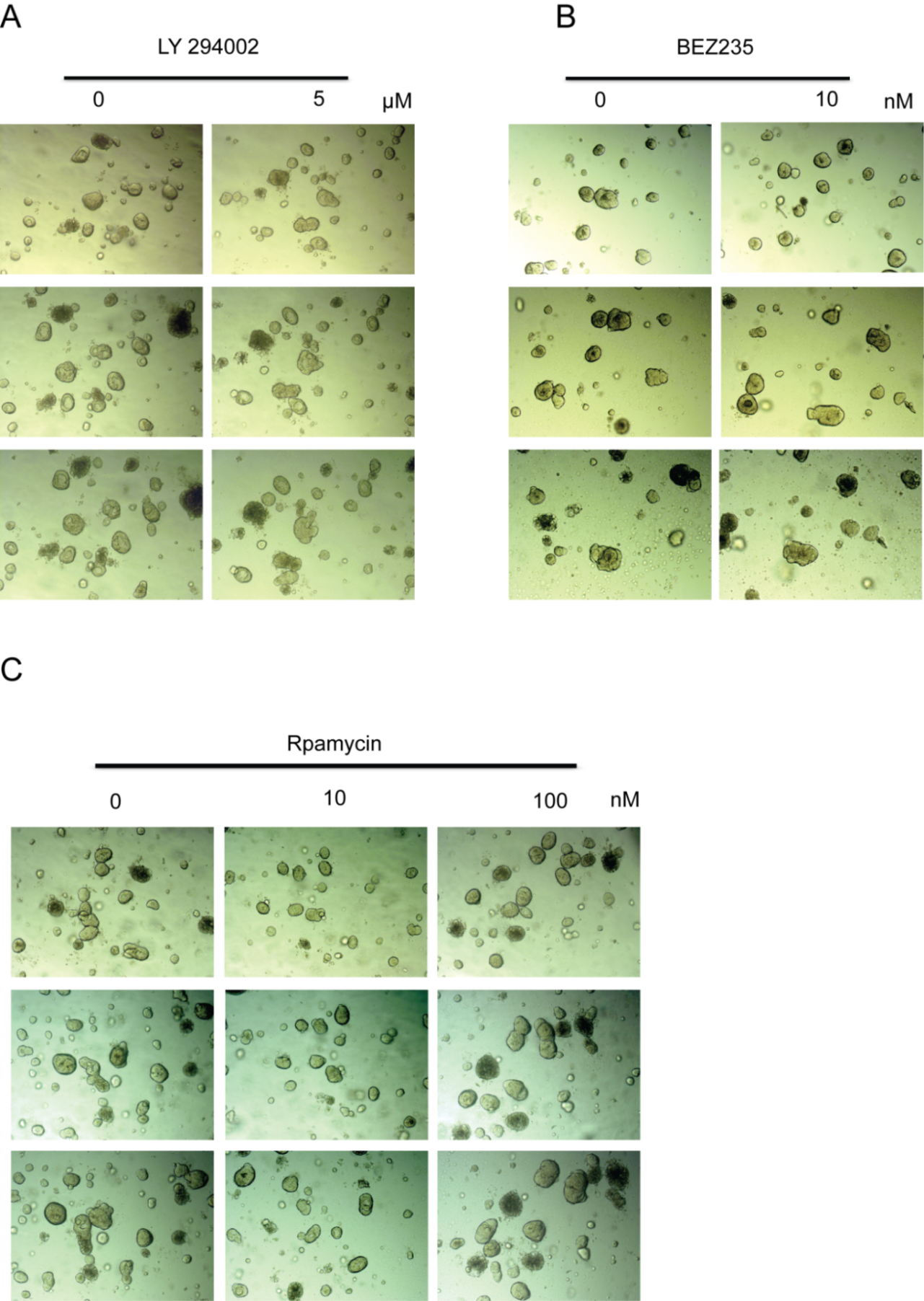


Figure S7 Effects of LY294002, BEZ235 and rapamycin on growth of human intestinal organoids. (A) Effect of LY294002 on growth of organoids (48 h). (B) Effect of BEZ235on organoids growth (48 h). (C) Effect of rapamycin on organoids growth (48 h).

**REFERENCE**

1. Yin Y, Bijvelds M, Dang W, Xu L, van der Eijk AA, Knipping K, Tuysuz N, Dekkers JF, Wang Y, de Jonge J, Sprengers D, van der Laan LJ, Beekman JM, Ten Berge D, Metselaar HJ, de Jonge H, Koopmans MP, Peppelenbosch MP, Pan Q. 2015. Modeling rotavirus infection and antiviral therapy using primary intestinal organoids. Antiviral Res 123:120-131.
